# Contrast enhanced ultrasound (*CEUS*) in blunt abdominal trauma

**DOI:** 10.1186/2036-7902-5-S1-S9

**Published:** 2013-07-15

**Authors:** Lucio Cagini, Sabrina Gravante, Corrado Maria Malaspina, Elviro Cesarano, Melchiorre Giganti, Alberto Rebonato, Paolo Fonio, Michele Scialpi

**Affiliations:** 1 Department of Surgical, Radiological and Odontostomatological Sciences, Thoracic Surgery, Perugia University, S. Maria della Misericordia Hospital, S. Andrea delle Fratte, 06134 Perugia, Italy; 2 Radiological and Odontostomatological Sciences, Complex Structure of Radiology, Perugia University, S. Maria della Misericordia Hospital, S. Andrea delle Fratte, 06134 Perugia, Italy; 3 Radiology Section. Health service. Navy Command of Brindisi, Brindisi, Italy; 4 Department of SurgicalSciences, University of Ferrara, Ferrara, Italy; 5 Institute of Diagnostic and Interventional Radiology, University of Turin, Turin, Italy

**Keywords:** Ultrasonography (US), Ultrasound contrast agent (USCA), Contrast-enhanced Ultrasound (CEUS), Computed Tomography (CT), Blunt abdominal trauma

## Abstract

In the assessment of polytrauma patient, an accurate diagnostic study protocol with high sensitivity and specificity is necessary. Computed Tomography (CT) is the standard reference in the emergency for evaluating the patients with abdominal trauma. Ultrasonography (US) has a high sensitivity in detecting free fluid in the peritoneum, but it does not show as much sensitivity for traumatic parenchymal lesions. The use of Contrast-Enhanced Ultrasound (CEUS) improves the accuracy of the method in the diagnosis and assessment of the extent of parenchymal lesions. Although the CEUS is not feasible as a method of first level in the diagnosis and management of the polytrauma patient, it can be used in the follow-up of traumatic injuries of abdominal parenchymal organs (liver, spleen and kidneys), especially in young people or children.

## Introduction

Ultrasonography (US) is highly sensitive in the assessment of the abdominal trauma, particularly in the detection of intra-abdominal fluid with a percentage varying from 63% to 99% [1.2]; the sensitivity of US is significantly reduced in the diagnosis of traumatic parenchymal lesions [2,3].

Currently, in the evaluation of patients with abdominal trauma, Computed Tomography (CT) is the reference standard [4]. The introduction in the clinical practice of Contrast-Enhanced Ultrasound (CEUS) has improved the sensitivity of the US in the detection and assessment of the extension of traumatic parenchymal lesions [1,5]. In addition, CEUS exceeded the limits of the B-mode US and the US Color and power-Doppler, and expanded the applications of the method especially in abdominal trauma in children.

The first results in the literature indicates the use of CEUS in patients with blunt abdominal trauma after the FAST (Focused Assessment with Sonography in Trauma) or the US, in hemodynamically stable patients with a history of low-energy trauma [1,4,6]. CT is reserved in cases of severe trauma, with clinical suspicion of multiorgan lesions and cases with inconclusive CEUS [6].

In the management of the polytraumic patient it is necessary to plan an effective, efficient and rapid diagnostic and therapeutic procedure, in order to reduce the morbidity and mortality of said patient.

The Authors assess the current role of CEUS in diagnosis of blunt abdominal trauma, analyzing limitations and advantages.

## CEUS: technique

The contrast agents eco-amplifiers are able to modify the acoustic impedance of tissues, interacting with ultrasound beams and increasing the echogenicity of the blood. The contrast media (CM) ultrasound (USCA, UltraSound Contrast Agent) consist of microbubbles containing inert gases and surrounded by membrane stabilizers.

The power of echogenic microbubbles and acoustic impedance depends on the size of the microbubbles. The microbubbles, unlike the tissues and the free gas, are not simply passive reflectors, but expand and compress in response to the stages of compression and rarefaction of the acoustic wave, with increasingly large hikes in diameter. The non-linear oscillation of microbubbles determines the emission of frequencies of said second harmonic with a frequency which is twice the insonation. Through the use of specific software, low acoustic pressures and an algorithm of specific processing, it is possible to selectively display the signals from the CM, separating the signal of the microbubbles from the one regarding the tissue. This particular signal is identified in real time by means of two main algorithms: Pulse Inversion (PI) and Contrast Pulse Sequence (CPS) [7,8].

The first generation contrast agents, consisted of air-filled microbubbles: they were particularly fragile and their quick and easy break involved a large inter-and intra-individual variation of the signal amplification with short duration of contrast effect.

The second-generation contrast agent (SonoVue - Bracco), used in our experience, is represented by inert gas-filled microbubbles and denser than air (sulfur hexafluoride) and delimited by membranes made of phospholipids stabilized, giving high strength and flexibility. Sulphur hexafluoride is eliminated via the respiratory system while the membrane phospholipids are metabolized in the liver. This CM is well tolerated (side effects-based incidence of anaphylactoid reactions have 0.001%), non-nephrotoxic [9] and with short half-life (approximately 12 minutes), but particular caution should be exercised in patients with cardiac and pulmonary disease [10]. These are characterized by strong power echogenic microbubbles in size sufficiently small (< 7 μm) to be able to pass through the capillaries but was unable to cross the endothelial fenestrations with persistence in the blood stream for a relatively long time [11-14]. In fact, unlike the CM used in CT or MRI, which spread rapidly in the extravascular interstitial space, the ultrasound contrast agent used in pharmacokinetics has the characteristic to remain confined to the vessel lumen, without spreading to interstitial level, and therefore are not filtered in the kidney. The CM is administered intravenously by bolus injection using needles 18-20 Gauge followed by a bolus of approximately 10 ml of saline solution [1].

## CEUS: pattern of enhancement

The CEUS technique involves the continuous insonation of the region of interest after the injection of CM with real-time and continuous evaluation of all the contrastrographic phases (arterial phase, venous phase, and late phase). The CEUS enhancement patterns in each phase are similar to that of CT or MRI; however, due to the different pharmacokinetics of the CM, the late phase of CEUS does not correspond to equilibrium phase as described for the extracellular CM used in CT [10]. The CEUS findings are related to the contrast material distribution and is defined as homogeneous, heterogeneous or absent. On the other hand, it is difficult to define the degree of the enhancement qualitatively when the parenchyma is considered.

The appearance of a normal abdominal parenchymal organ is homogeneous and hyperechoic in the absence of distortions of the echogenicity and vascular structures clearly distinguishable. Traumas can cause various parenchymal changes: bruises, lacerations, bruising, bleeding, heart attack or arteriovenous fistulas [1]. According to the mechanism of injury, bruises show different aspects, ranging from a simple edematous area, ill-defined with ultrasound contrast media, to hypoechoic areas characterized by reduced or absent perfusion. The lacerations appear as bands of linear or branched marked hypoechogenicity sharply defined, and the clinical course usually perpendicular to the surface of the organ (and dependent on the force lines of the trauma) and may be associated with capsular discontinuity of the profile. The intraparenchymal hematoma is valuable as a heterogeneously hypoechoic area with poorly defined contours in the context of which are not recognizable vascular structures; subcapsular hematoma appears as a lenticular area of absent enhancement surrounding parenchyma in which, if actively stocked, is recognizable extravasation CM. The spreading of contrast material within the peritoneal or retroperitoneal space is indicative of active bleeding [6,14]. The complete avulsion of the vascular pedicle of an organ (e.g. spleen or kidney) is realized with the complete absence of enhancement of the parenchyma in abdominal examination [6]. It is fundamental to the differential diagnosis of traumatic parenchymal lesions with other pathological conditions possible causes of hypoechoic in ultrasound with contrast medium: e.g. calcifications (clearly visible with conventional imaging), pseudoaneurysm, non-traumatic focal parenchymal lesions [15].

## CEUS: peritoneal trauma

The first diagnostic step in cases of abdominal trauma is the relief of payment intraperitoneal, indirect sign of parenchymal injury [4,5]. The hemoperitoneum is often associated with splenic injuries and/or liver and its size is usually related to the severity of the picture.

## Spleen

The spleen is the organ most frequently affected intra-abdominal trauma [3]. His exploration may be limited by the interposition of the coasts and the bloating of the splenic flexure, particularly at the level of inaccessible sites such as the upper pole and the phrenic sub region, especially in uncooperative patients (inability to vary the respiratory phases or decubitus).

**Figure 1:**
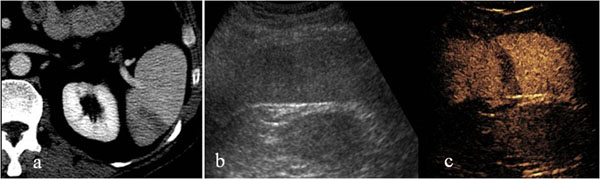
Spleen. Contrast-enhanced CT in venous phase (a), US-B-mode (b) and CEUS (c) in a 40 year- male patient with blunt abdominal trauma. CT shows a splenic hypodense parenchymal lacerative area (a) not recocognizable by US B-Mode examination (b). CEUS demonstrate splenic hypoechoic lesion corresponding to that of CT.

The splenic arterial phase of enhancement (early start at 12-18 seconds) has a relatively long duration with an aspect of organ uneven and called "zebra" (due to the movement of two circuits with red pulp and white pulp), which makes it difficult if not impossible the identification of tissue damage. Venous phase (approximately 40-60 seconds after i.v. contrast material injection) is accurate in the detection of traumatic lesions of the spleen (Fig. 1); in the venous phase the normal parenchyma presents a homogeneous contrast-enhancement of sufficiently long duration (about 5 - 7 minutes) [1,6,15]. Compared to the left kidney, which exhibits early enhancement but brief, the spleen appears less echogenic and hyperechoic during the arterial phase during the late phase.

Since the spleen acts as a filter for microbubbles, the splenic vein and its tributaries exhibit washout about 3 minutes after the start of contrast material [4], thus resulting in only a tenuous enhancement of the vessels due to entrapment intrasplenic. In this case, the venous vascular structures, soon hypoechoic, can create problems of differential diagnosis with perfusion defects parenchymal post-traumatic; fact, Valentine et al. recommend, when in doubt, a second evaluation with a second bolus of contrast agent [6].

Dose: for adults using a range between 0.6 and 1.2 ml. For children, the dose in ml is calculated using the following formula: age/20 [4].

## Liver

The liver is the second intraperitoneal parenchymal organ involved by trauma. CEUS examination, the arterial phase appears about 10-20 seconds after the injection of contrast material, lasts approximately 15 seconds and is quickly followed by the venous phase, which lasts about 2 minutes. The late phase lasts until complete clearance of the microbubbles from the liver parenchyma (4 - 6 minutes) and does not correspond to the equilibrium phase described for the extracellular contrast media used in CT or MR [10].

Dose: for adults using a range between 1.2 and 2.4 ml. For children, the dose in ml is calculated using the following formula: age in years/10. [4]

**Figure 2:**
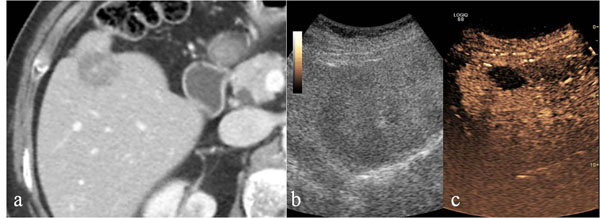
Liver. Contrast-enhanced CT in venous phase (a), US-B-mode (b) and CEUS (c) in a 67 year-old male patient with blunt abdominal trauma. CT shows a epatic hypodense intra-parenchymal traumatic area (a) not recocognizable by US B-Mode examination (b). CEUS demonstrate epatic hypoechoic lesion corresponding to that of CT.

CEUS in the healthy liver parenchyma shows diffuse and homogeneous hyperechoic in the absence of echotexture distortions. Traumatic injuries parenchymal are hardly appreciable examination U.S. [1] and occur at CEUS as areas of reduced or absent perfusion better highlighted during the late phase (Fig. 2).

The full exploration of the liver can be affected from the large surface of the liver to explore and limited to the study of areas not easily accessible (dome and lateral segments liver), particularly in uncooperative patients (inability to vary the respiratory phases or decubitus) by interposition of coasts and bloating stomach and intestines. In addition, the hematoma or laceration severe hepatic entity are not easily found in the acute phase as yet isoechoic compared with adjacent healthy parenchyma. For these reasons, the CT examination remains the imaging modality of reference, in particular in high-energy trauma.

### CEUS: retroperitoneal trauma

US is limited in the evaluation of retroperitoneal structures due to their depth and the interposition of the bowel [16].

Contrast-enhanced CT is the imaging modality of choice in diagnosis of retroperitoneal emergencies, providing important informations in the detection and the stage of parenchymal lesions and in definition the type, site and extent of abdominal fluid collections for a proper management [17,18].

### Pancreas

Pancreatic traumatic lesions are less frequent than spleen or liver ones, occurring in less than 2% of the cases. Since the high mortality and morbidity rates associated to these lesions, it is essential to come promptly and effectively to the diagnosis. Usually the pancreas is difficult to explore due to the interposed intestinal bloating. In literature very few studies reported low sensitivity and specificity of the US in cases of post-traumatic pancreatic damage [3], asserting the role of CT as a method of choice for the study of retroperitoneal organs. US examination may be useful to identify and detect peri-pancreatic collected fluid, when exploration is feasible. Therefore, currently, CEUS misses indication in the study of post-traumatic pancreatic lesions [19].

### Kidney

**Figure 3:**
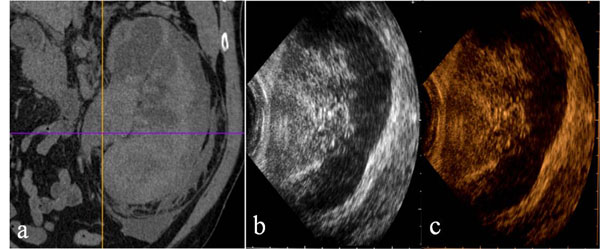
Kidney. Contrast-enhanced CT in venous phase (a), US-B-mode (b) and CEUS (c) in a 72 year-old female patient with blunt abdominal trauma. CT shows a renal subcapsular haematoma (a) recocognizable by US B-Mode examination (b).and CEUS corresponding to that of CT.

Renal trauma is relatively frequent and represents about 5% of the abdominal trauma [19].The kidneys show different degrees of enhancement in the cortex and the pyramids; the cortex almost immediately enhances very brightly and evenly, while the pyramids enhance diffusely from the periphery to the centre over about 30 seconds. The homogeneous phase of the kidneys generally lasts 2-2,5 min: this homogeneous phase (venous phase or nefrographic phase) is still the most effective for detection of traumatic injuries [1,6]. The recommended dose is the same as used for the study of the spleen (0,6 ml or ml of SonoVue: age in years/20). It is necessary to investigate the kidneys separately with two different boluses of contrast media [6]. The full exploration of both kidneys is usually hard: the left kidney is sometimes obscured by superimposed bowel gas and ribs on images from US evaluations [3]. Similarly to the liver and spleen, kidney contusion lesion can appear as an hypoechoic area without clear delimitation; laceration usually appears as a linear or branched hypoechoic band, perpendicular to the surface of the organ. A subcapsular hematoma appears as a nonhomogeneous collection surrounding the kidney. In the case of avulsion of the renal hilum, a total absence of parenchymal enhancement is found at CEUS. [1,6] (Fig. 3). The rapid-enhancement can generate questions of interpretation that can possibly be solved only with a second injection of contrast agent [17]. An injection of too high a dose of contrast media will have a negative effect due to the intense enhancement, potentially masking the presence of lacerations [6]. Moreover, as much as no microbubble excretion into urinary tract is found, CEUS evaluation detects only indirect signs (abdominal fluid) event of accidental bladder, ureter or collecting system failure. For the above limits, CT remains the method of choice for staging of renal damage [18].

### Adrenal

Traumatic lesions of the adrenal glands are extremely rare and often express a high-energy trauma therefore with high mortality or morbidity. US is particularly useful in emergency in children: an adrenal traumatic lesion can be realized in a heterogeneous nodular appearance with increased echogenicity compared with hepatic or renal parenchyma or as heterogeneous structural alteration of the adrenal region. However, the sensitivity of ultrasound is significantly lower compared to CT, currently considered to be gold standard for diagnosis of adrenal traumatic lesions [17]. CEUS can play an important role in the follow-up in selected patients, especially pediatric patients, to decrease radiation exposure.

### Retroperitoneal vessels

Traumatic injury of the abdominal aorta and retoperitoneal great vessels are infrequent but lethal due to the peritoneal or retroperitoneal rapid hemorrhage. The chances of survival increase with early intervention, so it is essential to get to diagnosis immediately. The retroperitoneum remains unknown to the FAST examination and poorly evaluated by US that underestimate free fluid [21-24]. Thus, the CT is the gold standard in case of abdominal aortic traumatic injuries due to the high sensitivity and specificity to detection and staging the injuries resulting useful for the choice of the appropriate treatment [25,26]. However, some studies assume a potential improvement of the diagnostic potential of CEUS in the case of aneurysm rupture for the ability to detect the extravasation of contrast agent [14,27].

### Diaphragm

Traumatic pulmonary hernia is a complication that may rarely be associated with blunt abdominal trauma. US can detect the interruption of the echogenic diaphragmatic band or of gaseous artifacts indicating digestive hernia and also it can identify the presence of hepatic or splenic parenchyma ectopic. US has a high number of false negative and is limited in estimating the extent of the lesions. At CEUS, as at B-mode US examination, the lesions of the diaphragm remain difficult to assess. In the emergency, CT is the gold standard for suspected diaphragmatic injuries due to the high spatial resolution and the ability to perform multiplanar reconstructions, which are essential for the diagnosis [24,28].

## Discussion

Ultrasonography (US) is a useful imaging modality in the detection of traumas, due to its high sensitivity for free intraperitoneal fluid. The execution is rapid even at the bedside. However, US does not show as much sensitivity for the detection of solid abdominal organs traumatic injury; furthermore, the absence of hemoperitoneum does not allow the exclusion of the presence of post-traumatic parenchymal injuries [29].

The introduction in clinical practice of contras-enhanced US (CEUS), increases the sensitivity and accuracy of the US; hovewer, the CEUS has some limitation in the evaluation the parenchymal injuries and retroperitoneum.

With respect to parenchymal injuries by CEUS, similarly to US, some locations (e.g. hepatic dome and the upper pole of the spleen) are poorly inaccessible. CEUS is enable to evaluate simultaneously abdominal parenchyma. In order to overcome this limitation, some authors propose to split contrast material in two or more boluses to study one or at most two ipsilateral parenchyma. According to this diagnostic strategy, the kidney must be the first organ to be studied because of the early and short enhancement peak (first two minutes), the same bolus (volume of about 2.4 ml) can be used for the study of the liver or the spleen in relation to the late reaching of a homogeneous enhancement (2 to 4 minutes after injection of contrast material) respectively for the abdominal quadrants of the right or left [1]. The study of the renal excretory cavity is not a limit to the examination of the urinary tract in the polytrauma patient, due to the lack of renal elimination of contrast material used in US.

With respect to retroperitoneal injuries similarly to US, the CEUS is limited by the interposition of the gastric and intestinal bloating, the constitutional habitus of the patient and in particular, the exploration of the retroperitoneum, which is sometimes impossible.

CEUS should be performed by operators at a high level competence. In fact administration of an excessive dose of contrast agent due to poor experience of the operator can affect the diagnosis: the dose depends on the target organ but also on the characteristics of the equipment used, the use of high doses of contrast agent results in excessive hyperechogenicity of the parenchyma which disturbs the identification of small traumatic injuries [7].

Additional limitations of the CEUS are the absence of three-dimensional scanning, the lack of whole-body exploration, extreme difficulty in detecting traumatic bowel and mesenteric lesions and the operator dependence that makes the technique "subjective". CT remains the imaging of the choice in trauma due to its high sensitivity and specificity [21,30,31] and the relatively non-invasive and rapid execution. In polytrauma patients, the use of CEUS was proposed as a first level examination, after the FAST, in order to reduce the number of CT examinations [1,4,6]. Because the US B-mode is able to demonstrate the presence of intra-abdominal fluid in most cases, but is poorly sensitive in detecting post-traumatic organ damage [3,15], some authors recommend the CEUS in complement of FAST or the US for the evaluation of liver, spleen and kidney trauma [10], others reserve the CEUS at low energy abdominal trauma. In splenic trauma Catalano et al. [15] use CEUS after US, when at US free fluid in the peritoneum is detected, in cases of doubt at US, in cases with persistent negative at US or laboratory-clinical suspicion of splenic injury, reserving CT examination to selected patients at a later time. Rhea et al. [32], although they consider appropriate the US for the detection of hemoperitoneum, they consider the CT ad an imaging technique with a significant positive impact for the management of trauma patients to reduce the mortality. Concerning pediatric patients, Benya et al. [29] argue that the US does not exclude negative organ damage, in addition, US examination is not sufficiently helpful in planning a subsequent possible surgical treatment. In pediatric patients, the examination has greater sensitivity and reference in the management of trauma is contrast-enhanced CT [17]. In polytrauma patients, to avoid diagnostic step that could result in the loss of valuable time and the delay of therapeutic maneuvers, the use of a prompt diagnostic protocols that ensures high sensitivity, specificity and diagnostic accuracy are need [33-35]. In the assessment of post-traumatic abdominal injuries, literature studies show the sensitivity of the FAST and the US of 56% and 68% respectively, with no recognizable organ damage in a percentage variable between 16 % and 35% compared to CT [36]. CEUS is an accurate diagnostic tool that should not be considered as an alternative to CT but rather as a supplement to conventional technique useful in cases of inclusive CT [37,38].

In the low-energy trauma and in hemodynamically stable patients, the US can be used as a first-level examination; when US detect intra-abdominal fluid CT examination is need. In the high-energy trauma the use of US as first line diagnostic is superfluous and damaging and the use of CT without and with i.v.c onstrast material is imperative. In order to reduce the radiation dose, particularly in young people or children, CEUS has an important role in the follow-up of conservatively treated traumatic injuries of the abdominal parenchymatous organs (liver, spleen and kidneys) diagnosed by CT [39,40].

## Competing interests

The authors declare that they have no competing interests.

## Declarations

This article has been published as part of *Critical Ultrasound Journal* Volume 5 Supplement 1, 2013: Topics in emergency abdominal ultrasonography. The full contents of the supplement are available online at http://www.criticalultrasoundjournal.com/supplements/5/S1. Publication of this supplement has been funded by the University of Molise, University of Siena, University of Cagliari, University of Ferrara and University of Turin.
